# Determination of the relationship between major histocompatibility complex alleles and childhood onset obsessive-compulsive disorder

**DOI:** 10.3906/sag-2103-224

**Published:** 2021-12-09

**Authors:** Ümit LÜLEYAP, Gökhan KARACAOĞLAN, Ayşegül Yolga TAHİROĞLU, Akgün YAMAN, Perçin PAZARCI, Salih ÇETİNER, Davut ALPTEKİN, Yaşar SERTDEMİR, Gülşah EVYAPAN, Doğa LÜLEYAP

**Affiliations:** 1 Department of Medical Biology and Genetics, Faculty of Medicine, Çukurova University, Adana, Turkey; 2 Department of Child and Adolescent Psychiatry, Faculty of Medicine, Çukurova University, Adana, Turkey; 3 Department of Medical Microbiology, Faculty of Medicine, Çukurova University, Adana, Turkey; 4 Department of Immunology, Faculty of Medicine, Çukurova University, Adana, Turkey; 5 Department of Biostatistics, Faculty of Medicine, Çukurova University, Adana, Turkey; 6 Department of Pediatric Intensive Care, İzmir Health Sciences University Tepecik Education and Research Hospital, Izmir, Turkey

**Keywords:** Autoimmunity, children, MHC, neuropsychiatric disorders, OCD

## Abstract

**Background/aim:**

About half of the cases of obsessive-compulsive disorder (OCD) occurring in childhood/adolescence occur with similar symptoms both in childhood and adulthood. Immunologic stress is claimed to be a risk factor in the etiology of childhood onset OCD. Our aim was to elucidate the relationship between childhood onset OCD risk and MHC complex I and II alleles.

**Materials and methods:**

MHC alleles of 49 OCD children together with 277 healthy children (aged 4–12) were analyzed by PCR. Results were evaluated by using univariate analysis and multivariate logistic regression analysis.

**Results:**

A2, A29, C4, DRB3.1, and DRB1*16 alleles were found to increase the risk of OCD.

**Conclusion:**

The relationship found between DRB locus and OCD in this study was remarkable since there have been studies on different populations reporting similar relationship between DRB locus and rheumatoid arthritis, which is also an AID. MHC class I and class II alleles were found to increase the risk of OCD in our study, which serves as a suitable model for studies suggesting that MHC genes do not work completely independently. Even though the MHC class I and II genes are considered to have different roles in immune response, in fact they tend to work in cooperation. As in previous studies on AIDs, there is a linear relationship between MHC class II alleles and OCD risk.

## 1. Introduction

Obsessive-compulsive disorder (OCD) was first defined in psychiatric literature by Esquirol in 1838 [1]. OCD, which was first thought as a symptom of depression, is one of the few psychiatric disorders whose diagnostic criteria did not change much since Freud [2]. OCD started to be accepted as a distinct clinical disorder at the beginning of the 20th century. OCD is characterized by prominent discomfort in a person’s life caused by recurring obsessions and compulsions. In addition, it is a health problem, which causes waste of time, disturbs a person’s daily routines, occupational or educational functionality and social interactions. In DSM-5, published in May 2013, OCD was removed from being classified under the name of anxiety disorders and classified under a new title, “obsessive-compulsive disorder and related disorders” [3].

In our study, it was determined that the gender difference in childhood OCD patients was not significant. However, in a similar study covering childhood and adulthood, it was shown that the difference between the sexes was more common in women in adulthood [4].

Studies that aim to determine the etiology of OCD (a heterogenic disorder) accelerated together with developing genetic studies and brought us new insights to understand the causes of OCD. Determination of the relationship between OCD and genetic-induced diseases such as Tourette syndrome (TS) and chronic motor twitching, recognition of OCD spectrum disorders, determination of the relationship between OCD and serotonin, determination of brain metabolics, changes in OCD and the objectification of OCD diagnosis with the DSM scale have given prestige to genetic studies and it has directed researchers to this field [5].

OCD is defined as a neurobiological disorder that has multifactorial etiology. Lately, immunologic stress is claimed to be a risk factor in the etiology of OCD with childhood onset. Normally, immune system recognizes healthy cells of the body and distinguishes them from foreign antigens. Autoimmune diseases (AIDs) arise, when a change occurs that results in failure of this recognition process [6].

Human leukocyte antigen (HLA) genes are first defined in white blood cells and misnamed after it, however, after the determination of this antigenic structures in all tissues they are named as major histocompatibility complex (MHC) [6]. MHC gene region, which encodes tissue antigens for the immune system to distinguish between healthy cells and foreign antigens, is an important element of autoimmune response. Exogenous antigens that have molecular similarities to tissue antigens are known to cause autoimmune response by molecular mimicry [7]. Also, some haplotypes of MHC system are recognized to trigger autoimmune response in targeted tissues/organs by apoptosis and cause cell loss [8].

There are personal differences in susceptibility to AIDs, allergy, infection or various malign diseases seen in childhood. This situation is related to genetic diversity based on MHC polymorphism along with environmental factors [9].

There are not many studies on the relationship between psychiatric disorders and MHC genes. Few studies report the relationship between TS and MHC genes, in which A11, A26 alleles are found as risk factors and A13, A24 alleles are found as protective [10]. Psychiatric disorders and their relationship with MHC genes are mostly studied in schizophrenia. DR1 allele is determined to be found more frequently in Japanese and Turkish patients diagnosed with schizophrenia [11]. According to a meta-analysis, alleles related to schizophrenia are A9, A10, A24, A28, DRB1 and protective alleles are DRB1–4, DQB1–6 [12].

In this study, we aimed to research the relationship between childhood onset OCD and MHC alleles.

## 2. Materials and Methods

This study includes 49 children patients, referred to Çukurova University Faculty of Medicine, Department of Psychiatry. Patients were diagnosed with rapid onset of OCD or tics, together with 277 healthy children.

When sample size analysis for 20% exposure rate with values, Type 1 error = 0.05 power = 0.8 and reference OR = 2.5, the number of patients to be reached was found to be 50 (Rodriguez et al., 2017). The patient/control ratio has been accepted as 5/1 and it is planned to reach a minimum of 250 healthy people. Thus, 49 patients and 277 healthy children were included in the study.

All children included in the study are aged between 4–12. Children that have frequent upper respiratory tract infection, acute rheumatic fever, rheumatic heart disease, OCD or TS were not included in control group. Study was approved by the human studies ethical committee of the Çukurova University and informed consent was obtained from all subjects and their parents.

Psychiatric diagnoses of the children were evaluated by psychiatry specialists according to the DSM-V by using Washington University in St. Louis Kiddie Schedule for Affective Disorders and Schizophrenia (WASH-U-KSADS). OCD symptom scale was determined by using Children’s Yale-Brown Obsessive-Compulsive Scale (CY-BOCS).

DNA was isolated from blood. DNA samples of the patient and control groups were prepared on the GenoM-6 genovision device using Qiagen’s isolation kit. LABType^tm^ SSO (site specific oligo nucleotide) technique was applied using One Lambda kit (Thermo Fischer Inc.). A suspension array platform, fluorescently encoded microspheres were used as a solid support to immobilize the oligonucleotide probes. Target DNA is amplified with biotinylated, locus-specific primers. The amplified product is then denatured and hybridized with oligonucleotide probes. The amplified product hybridized to microsphere-bound oligonucleotide probes which was labeled with R-Phycoerythrin Conjugated Streptavidin (SAPE) and subsequently processed by LABScan. The assay was performed in a single well of a 96-well PCR plate.

Ages, obsession/compulsion scores, severity scores and antistreptolysin O (ASO) values of patients were evaluated with OCD given in Table 1. Aggravation of OCD symptoms was observed in all patients.

### 2.1. Statistical analysis

SPSS 20 program was used in the analysis of the data. (IBM Corp. Released 2011. IBM SPSS Statistics for Windows, Version 20.0 Armonk, NY: IBM Corp.). Data are expressed as arithmetic mean, standard error of mean, and number. Parametric tests were used for data with normal distribution, and nonparametric tests were used for qualitative data and the data that did not show normal distribution. Fisher’s chi-square test and Backward logistic regression analysis were used in the analysis of the data. For the alleles included in the logistic regression model, the reference category was determined as subjects without the allele, and the risk category was determined as subjects with the allele (homozygous or heterozygous). A value of p < 0.05 was considered statistically significant.

## 3. Results

First, Fisher’s chi-square test is applied to the results and MHC alleles, whose p values are smaller than 0.20, are found (as shown in Table 2). Then, Backward logistic regression analysis is performed to these alleles and protective/risk factor alleles are determined (as shown in Table 3). According to Backward logistic regression analysis, alleles whose value is lower than 1 are evaluated as protective (reducing risk) and alleles whose value is higher than 1 are evaluated as risk factors (increasing risk).

## 4. Discussion

OCD is a neuropsychiatric disorder, which is heterogenic due to phenotypic diversity affecting nearly 2% of the world population [3]. Although definite etiology is unknown, there are studies showing that genes associated with OCD and environmental factors together constitute a risk [13]. Importance of neurobiology in the etiology of OCD is revealed by various studies. Thus, pharmacological and neurobiological studies determined the role of neurotransmitters in OCD and in similar disorders [14]. In line with the results obtained from these studies, dopamine and serotonin hypotheses were generated stating two different enzymes, COMT and MAO-A, play a role in the metabolism of biological amines functioning in the synaptic transmission [15–17]. Moreover, results obtained from twin studies also revealed the importance of genetic factors.

The relationship between complex diseases caused by autoimmunity and HLA dates back to 50 years ago. However, the relationship between HLA, OCD, and PANDAS is based on the work of Swedo in 1989 [18,19]. Allen, Leonard, and Swedo introduced their hypothesis based on A/B-hemolytic streptococcus infections related with the start and exacerbation of OCD symptoms [20,21].

MHC genes are responsible for antigen presentation to T cell receptors. These genes are divided into MHC-I genes involved in endogenous antigen presentation and MHC-II genes responsible for the presentation of infectious exogenous antigens [22].

Recent genomic-scale exome-sequencing studies have shown that SNP polymorphisms in the MHC locus play an important role in the immunological pathways on antigen presenting and processing as well as in the etiology of OCD disease [23,24]. In addition, studies on MHC and infectious diseases have shown that MHC class II alleles; HLA-DRB1 and HLA-DQB1 have also been associated with more than 100 infectious diseases.

Previous studies have shown that there was a close relationship between OCD and the DRB1 alleles of MHC-II. Recent studies support the role of HLA class II alleles (DRB1) in the central nervous system in the pathophysiological model of OCD (Natalia Rodriguez, 2019). In our study, however, we found that in addition to DRB1 alleles HLA-DRB 3.1 was also related to OCD. The possible reason why we found a relationship between HLA-DRB 3.1 and OCD originates from the fact that the only difference between DRB1 and DRB3.1 alleles is the conversion of valine amino acid to glycine in the 86th position (86 Val----Gly) [25] inferring that they are almost identical in their nature. Therefore, our findings for HLA-DRB 3.1 are quite remarkable when considering this amino acid conversion.

HLA-DRB1 and -DRB3 are two separate genes that differ in the HLA-DR beta chain. It was also found that DRB1 gene was expressed at a higher level due to the regulatory sequences located proximal to the HLA-DRB locus, which is highly polymorphic compared to the HLA-DRB3 locus [26].

The risk of OCD in patients, who had two copies of the DRB3 allele was found to be 86 times higher compared to controls with one copy according to statistical evaluation. DRB3.1 allele appears to be riskier than DRB1 since there are no homozygous cases carrying the DRB1 allele in the patient group. However, when we compare the DRB1 and DRB3.1 alleles as single copies in terms of OCD risk, the risk of heterozygotes with DRB3.1 alleles was 2.7, while those with DRB1 alleles had a risk of 6.25. In conclusion, it can be inferred that DRB1 and DRB3.1 alleles are important risk factors for OCD, and DRB1 along with DRB3.1 alleles are compatible with our OCD expectation.

In our study, MHC-class I alleles; A2, A29, and C4 were also found to be risk factors for OCD. In our analysis of where this mismatch does not fit, we concluded that heterozygous individuals with the DRB1 and DRB3.1 alleles in the patient group had MHC-I alleles at different rates. As a matter of fact, it was determined that 12 of the 20 patients carrying the DRB3.1 allele were homozygous, while 8 were heterozygous. From these heterozygous patients; 37.5% had A2, 25% had A29, and 12.5% had C4 alleles together with DRB3.1 allele. In addition, of the 10 heterozygous individuals carrying a single copy of the DRB1 allele in the patient group; 30% had A2, 20% had A29, and 30% had C4 allele. Although HLA-A, B, and C are located in the HLA class I regions, they exhibit linkage disequilibrium (LD) with the class-II encoded DR/DQ haplotypes. The presence of this strong LD with the HLA class II region has made it difficult to identify independent effects for the class I region with any AIDs [27].

Furthermore, a new study indicating the strong relationship between multiple sclerosis and MHC locus DRB and C in the European population was remarkable for showing the importance of DRB and C locus in autoimmune related diseases like OCD [28].

In addition, C4 allele belonging to (residing) C locus was found to increase the OCD risk 9.88 times when it is double copy in our study. There have been also an important study showing the decreased risk of rheumatoid arthritis associated with C locus polymorphisms [29]. Although OCD is different from rheumatoid arthritis, it is important for showing the role of similar alleles and locus on autoimmune based diseases.

Despite a recent study in a different population suggested that the effects of alleles in DRB and C locus were independent of each other on rheumatoid arthritis, our findings regarding the association between DRB and C locus, two important elements of autoimmunity, in Turkish OCD population is remarkable [30]. Also, Simmonds et al. found a strong association between DRB and C locus for Graves’ disease which also supported our OCD results [31].

AID and MHC class-I region association has been detected for; MHC-B with type 1 diabetes and MHC-C with multiple sclerosis and Graves’ disease, which provides further evidence of a possible role for infection in AID onset [9].

MHC alleles increasing the OCD risk found in our study belong to different classes, which serve as an appropriate model for studies suggesting MHC genes do not work completely independently. However, condensation of alleles that are increasing the risk of OCD in MHC class-II in our study also coincides with previous evaluations made on AIDs [31].

In conclusion, although our study showed that some of the MHC alleles increase the risk of OCD, more research has to be done on this issue and on the relationship between MHC polymorphisms and AIDs to attain conclusive results.

## Figures and Tables

**Table 1:** Ages, obsession/compulsion scores, severity scores, and ASO values of patients (mean ± S.E.M, n = 49).

Age	Compulsion score	Obsession score	Severity score	ASO (mg/dL)
10.143 ± 0,309	12.245 ± 0.473	11.97959184 ± 0.604	3.327 ± 0.107	207.898 ± 28.930

**Table 2:** Results of Fisher’s chi-square test.

Allele	Allele count	Control	OCD	p value
A2	0	188	31	0.13
	1	79	13	
	2	10	5	
A3	0	227	36	0.14
	1	45	10	
	2	5	3	
A24	0	192	32	0.20
	1	73	17	
	2	12	0	
A29	0	265	43	0.03
	1	12	6	
	2	0	0	
B7	0	261	43	0.03
	1	16	5	
	2	0	1	
B15	0	255	42	0.15
	1	22	7	
	2	0	0	
B49	0	249	47	0.18
	1	28	2	
	2	0	0	
B51	0	216	43	0.19
	1	57	5	
	2	4	1	
C3	0	246	48	0.05
	1	30	1	
	2	1	0	
C4	0	185	32	0.18
	1	86	11	
	2	6	6	
DQ2	0	202	35	0.13
	1	64	14	
	2	11	0	
DRB1*4	0	195	41	0.04
	1	70	8	
	2	12	0	
DRB1*7	0	227	44	0.13
	1	43	5	
	2	7	0	
DRB1*8	0	260	46	0.05
	1	17	2	
	2	0	1	
DRB1*13	0	241	37	0.07
	1	34	12	
	2	2	0	
DRB1*14	0	244	40	0.08
	1	32	7	
	2	1	2	
DRB1*16	0	257	38	0.01
	1	19	10	
	2	1	1	
DRB3.1	0	240	29	0.00
	1	35	8	
	2	2	12	
DRB3.2	0	139	34	0.01
	1	100	13	
	2	38	2	
DRB5.2	0	262	43	0.05
	1	14	5	
	2	1	1	

**Table 3:** Results of Backward logistic regression analysis.

Allele	Allele count	Odds ratio	p value
A2	0	reference	-
	1	1.611	0.266
	2	5.683	0.013
A29	0	reference	-
	1	4.611	0.019
	2	-	-
C4	0	reference	-
	1	0.961	0.930
	2	9.880	0.001
DRB3.1	0	reference	-
	1	2.738	0.036
	2	86.259	0.000
DRB1*16	0	reference	-
	1	6.251	0.000
	2	-	-
